# Integration of Solexa sequences on an ultradense genetic map in *Brassica rapa *L.

**DOI:** 10.1186/1471-2164-12-249

**Published:** 2011-05-19

**Authors:** Wei Li, Jiefu Zhang, Yanglong Mou, Jianfeng Geng, Peter BE McVetty, Shengwu Hu, Genyi Li

**Affiliations:** 1 College of Agronomy, Northwest A&F University, Yangling, Shaanxi, 712100, China; 2 The Department of Plant Science, University of Manitoba, R3T2N2, Canada; 3 Jiangsu Academy of Agricultural Sciences, Nanjing, China; 4 Baylor College of Medicine, Houston, 77030, USA

## Abstract

**Background:**

Sequence related amplified polymorphism (SRAP) is commonly used to construct high density genetic maps, map genes and QTL of important agronomic traits in crops and perform genetic diversity analysis without knowing sequence information. To combine next generation sequencing technology with SRAP, Illumina's Solexa sequencing was used to sequence tagged SRAP PCR products.

**Results:**

Three sets of SRAP primers and three sets of tagging primers were used in 77,568 SRAP PCR reactions and the same number of tagging PCR reactions respectively to produce a pooled sample for Illumina's Solexa sequencing. After sequencing, 1.28 GB of sequence with over 13 million paired-end sequences was obtained and used to match Solexa sequences with their corresponding SRAP markers and to integrate Solexa sequences on an ultradense genetic map. The ultradense genetic bin map with 465 bins was constructed using a recombinant inbred (RI) line mapping population in *B. rapa*. For this ultradense genetic bin map, 9,177 SRAP markers, 1,737 integrated unique Solexa paired-end sequences and 46 SSR markers representing 10,960 independent genetic loci were assembled and 141 unique Solexa paired-end sequences were matched with their corresponding SRAP markers. The genetic map in *B. rapa *was aligned with the previous ultradense genetic map in *B. napus *through common SRAP markers in these two species. Additionally, SSR markers were used to perform alignment of the current genetic map with other five genetic maps in *B. rapa *and *B. napus*.

**Conclusion:**

We used SRAP to construct an ultradense genetic map with 10,960 independent genetic loci in *B. rapa *that is the most saturated genetic map ever constructed in this species. Using next generation sequencing, we integrated 1,878 Solexa sequences on the genetic map. These integrated sequences will be used to assemble the scaffolds in the *B. rapa *genome. Additionally, this genetic map may be used for gene cloning and marker development in *B. rapa *and *B. napus*.

## Background

There are several PCR-based molecular marker detection methods such as amplified fragment length polymorphism (AFLP), random amplified polymorphic DNA (RAPD), simple sequence repeats (SSR) and sequence related amplified polymorphism (SRAP) that are commonly used in molecular marker development, genetic mapping, genetic diversity analysis and high density genetic map construction [1]. Compared with other PCR-based molecular marker detection methods, SRAP is more flexible and easier to use. It produces similar fragment size range and number of polymorphic loci as AFLP, but the SRAP protocol is much simpler than the AFLP protocol. Unlike AFLP, there is no limitation of SRAP primers and primer combinations since one forward primer can be combined with unlimited reverse primers to have as many primer combinations as needed. Compared with RAPD, SRAP primers amplify more loci and are more reproducible. In contrast, one pair of SSR primers is normally targeted for one or a few loci in a gene genome, while a pair of SRAP primers in a PCR reaction produces several or even more than a dozen of polymorphic loci.

SRAP has a broad range of applications in genomic analysis. In particular, SRAP is a feasible technology for constructing high density and even ultradense genetic maps. For example, Sun et al. [2] constructed an ultradense genetic map containing 13,351 SRAP markers in *B. napus*, which is the most saturated map in Brassica species that has ever been constructed. Moreover, SRAP is an effective method for map-based gene cloning and molecular marker assisted selection (MAS). With SRAP markers, Zhang et al. [3] cloned the first gene that controls seed coat color in *B. rapa*. In cucumber, SRAP markers that are linked to warty fruits and sex type were developed [4,5]. A Fusarium resistance gene was tagged with SRAP markers in eggplant [6]. One SRAP marker associated with grain Cd concentration was found in oat [7]. In association mapping, four SRAP markers were identified to associate with freezing tolerance in alfalfa [8]. Moreover, SRAP is very effective to study genetic diversity since it does not need genome sequence information. For examples, genetic diversity and hybrid identification in Paeonia was analyzed with SRAP markers [9]. Diversity of buffalo grasses was compared using SRAP markers and the results suggested that polymorphic SRAP loci are abundant in this species [10]. Genetic diversity of Cynodon accessions was also assessed by SRAP [11]. In broccoli, SRAP was used to estimate genetic similarity among parents of hybrids [12]. Additionally, SRAP was adequate to perform QTL analysis, which was demonstrated in *B. napus *[13].

Next generation sequencing technology dramatically increases sequencing throughput. It is possible to sequence 1000 genomes in a few years instead of decades [14,15]. In soybean, next generation sequencing was used to sequence a reduced representation library to find large number of SNPs and 1,790 SNPs used to construct a high resolution genetic map that allowed the assembly of 97% sequence scaffolds into a genome sequence [16]. In maize, Sequenom-based SNP-typing assay was used to identify 1,359 SNPs in the transcriptome and 75% of these SNPs were confirmed and applied in association analysis [17]. Moreover, Illumina's sequencing was directly used in high throughput genotyping in rice and identification of breakpoints of recombination and gene mapping was conducted using the next generation sequencing approach [18]. Therefore, next generation sequencing is being extensively used in genome sequencing, gene identification and gene expression profiling.

In this report, Illumina's Solexa sequencing technology was used to sequence tagged SRAP products and 1,878 SRAP polymorphic loci with known Solexa paired-end sequences integrated on an ultradense genetic map in *B. rapa*. Eventually over 10,000 polymorphic loci were assembled in the genetic map. These sequenced polymorphic loci might be used for the assembly of genome sequence. Moreover, the ultradense genetic map is very powerful in molecular marker development, QTL analysis and map-based gene cloning.

## Methods

### Plant materials and DNA extraction

A yellow seeded *B. rapa *variety 'Yellow Sarson' and a Chinese cabbage doubled haploid (DH) line 'RI16' were crossed to develop a recombinant inbred (RI) line mapping population. The F_1 _plants were selfed to obtain F_2 _seeds and over 500 F_2 _plants were produced. From F_2_, the single seed descent (SSD) method was used to produce the following generations. Ninety-two F_7 _RI lines and the parental lines were selected for Illumina's Solexa sequencing and genetic map construction (Additional file 1). The DNA was extracted from leaves with a CTAB method as described previously [2].

### SRAP PCR

Three sets of SRAP primers (Additional file 2) were included to produce SRAP products. The SRAP PCR running program was the same as described by Li and Quiros [19] and SRAP PCR was set up as described by Sun et al. [2]. SRAP PCR reactions were performed in a 10 μl mixture containing 50 ng of genomic DNA, 375 μM dNTP, 0.15 μM of each primer, 1× PCR buffer, 1.5 mM MgCl_2 _and 1 unit of Taq polymerase. The SRAP PCR running program was 94°C for 3 min, 5 cycles of 94°C for 1.0 min, 35°C for 1.0 min, 72°C for 1.0 min, followed by 30 cycles of 94°C for 50 sec, 50°C for 50 sec, 72°C for 50 sec and final extension 72°C for 10 min.

The first set of SRAP primers consisted of fourteen forward primers (Set1F01-Set1F14) and 192 reverse primers (Set1R001-Set1R192), most of which were previously used in the construction of an ultradense genetic map in *B. napus *[2]. Only the DNA of two parental lines was amplified with these primers in 14 384-well plates. In each 384-well plate, 2 DNA samples, one forward primer and 192 reverse primers were included to produce 384 PCR reactions. In total, 5,376 SRAP PCR reactions were set up with 2,688 SRAP primer pairs in 14 384-well plates.

The second set of SRAP primers consisted of four forward primers (Set2F01-Set2F04) and 96 reverse primers (Set2R01-Set2R96). These forward primers shared 12 identical nucleotides (5'-GAGTCCAAACCG-3') at the 5' end while all 96 reverse primers had another 12 nucleotides (5'-CGCAAGACCCAC-3') at the 5' ends. These four forward primers were combined with these 96 reverse primers to form 384 SRAP primer pairs. Ninety-four DNA samples were amplified with these 384 primer pairs in 94 384-well plates respectively.

The third set of SRAP primers consisted of one forward primer (Set3F01) and 384 reverse primers (Set3R001-Set3R384) that were combined to obtain 384 SRAP primer pairs. The forward primer was used previously and the reverse primers were selected from the primer collections used for gene cloning in the lab. The same PCR set-up was performed as in the second set of SRAP primers. Similarly, 94 384-well plates were used to amplify all ninety-four DNA samples.

### Tagging of SRAP PCR products

The SRAP products from the first round of PCR were tagged in the second round of PCR with tagging primers.

To tag the SRAP PCR products obtained with the first set of SRAP primers, two 12 nucleotide heads (5'-GAGTCCAAACCG-3' and 5'-CGCAAGACCCAC-3') were joined respectively with the tails that consist of 11~14 nucleotides located at the 5' ends of the 14 forward SRAP primers to form tagging primers Set1T01-Set1T28 (Additional file 2). These 14 pairs of tagging primers were used to tag SRAP PCR products amplified with the two parental lines respectively, which allowed recognizing Solexa sequence originality. All tagging primers were used as forward primers and combined with the same 192 SRAP reverse primers (Set1R001-Set1R192) to amplify the SRAP PCR products in a specific PCR program.

To tag the SRAP PCR products obtained with the second set of SRAP primers, ninety-four tagging primers Set2T01-Set2T94 (Additional file 2) were designed for the 94 384-well plates in SRAP PCR. Since four forward primers of the second SRAP primers set had 12 identical nucleotides (5'-GAGTCCAAACCG-3') at the 5' ends, these nucleotides were used as tails and combined with ninety-four nine nucleotide heads to form ninety-four tagging primers. The tagging primers were used as forward primers. A 12 nucleotide (5'-CGCAAGACCCAC-3') primer that was shared by all ninety-four SRAP reverse primers in the second set of SRAP primers was used as reverse primer.

To tag the SRAP PCR products amplified with the third set of SRAP primers, another ninety four tagging primers were designed for the ninety-four 384-well plates in SRAP PCR. The first 12 nucleotides (5'-CGCAAGACCCAC-3') at the 5' end of the only SRAP forward primer was used as common 3' tails of tagging primers and ninety four different nine-nucleotide heads were added at the 5' end of the only SRAP forward primer to form ninety-four tagging primers (Set3T01-Set3T94). These tagging primers were used as forward primers and combined with the same 384 SRAP reverse primers.

The tagging PCR reactions were performed in a 10 μl mixture containing 0.2 μl of SRAP PCR products that were added with a 384-pin replicator and other components were the same as those in SRAP PCR. Tagging PCR was run with a specific PCR program: 94°C for 3 min, followed by 30 cycles of 94°C for 1.0 min, 50°C for 1.0 min, 72°C for 1.0 min and final extension 72°C for 10 min.

All SRAP primers and tagging primers were list in Additional file 2.

### Pooled PCR products for Illumina's Solexa sequencing

All tagged PCR products in all 202 384-well plates were pooled and one round of size selection was performed. The PCR products with approximately 100 to 400 bp size were collected from an agarose gel and purified with Qiagen spin filter columns.

Illumina's Solexa sequencing of the pooled sample was performed by BGI (Shenzhen, China) and 13.8 million paired-end sequences (1.25 GB sequence) were obtained [GenBank:SRA035245].

### Data analysis of Solexa paired-end sequences

To count how many Solexa paired-end sequences in a sequence file (text file), the sequence file was loaded in the Ubuntu Linux operation system. The 'word count' (wc) function was used to count the number of lines contained in the sequence file. Since every four lines in the sequence file contained one paired-end sequence, the total number of paired-end sequences was calculated.

Brute-Force Algorithm was used in a Java program to find the primer sequences in each paired-end sequence. With a maximum of one mismatch allowed, the primers were identified in the first 25 nucleotides of the sequences. The identified primer sequences were then removed from the sequences and the primer names were assigned to the paired-end sequences. Since all the sequences were paired-end ones, only those in which both forward and reverse primers were found were included in the following analysis.

The sequences were sorted and assigned to different files based on the forward primers. Therefore, the large sequence files were divided into 216 files that corresponded to all tagging primers. These 216 files were classified into three groups that corresponded to three sets of SRAP primers. These three groups were analyzed respectively. Java programs were developed to count the duplicates of every unique Solexa paired-end sequence in each file.

### Construction of an ultradense genetic map

To integrate Solexa sequences on a genetic map, SRAP technology was used to construct an ultradense genetic map. In total, 805 SRAP primer pairs were used to run the mapping population. Six hundred and eighty-two out of 3,072 pairs from the first and third sets of SRAP primers used for preparing Illumina's Solexa sequencing sample were selected. Two hundred and forty-one SRAP primer pairs corresponded to those used in the construction of a previous genetic map of *B. napus *[2] and 175 out of 241 SRAP primers overlapped with the above 682 SRAP primer pairs. Additionally, fifty-five SRAP primer pairs from forward primer 'ME2' (5'-TGAGTCCAAACCGGAGC-3') and other forward primers were combined with reverse primers of the first and third sets of SRAP primers.

The same RI line population in Illumina's Solexa sequencing was used for mapping. All forward primers were fluorescently labelled. The set-up of SRAP PCR reactions and running program were the same as those used in the first round of SRAP PCR for preparing Illumina's Solexa sequencing samples. The PCR products were separated with an ABI 3100 Genetic Analyzer (Applied Biosystems, California, USA). The data were first analyzed with ABI's GenScan software and then loaded into Genographer software for scoring polymorphic loci.

JoinMap version 3.0 Software was employed to construct a genetic map. Since there were thousands of SRAP markers, all markers were first classified into 10 groups at a high LOD score. Second, each group was divided into parts of which each has less than 200 markers. Third, a third round map for each part was generated and closely linked markers with a genetic distance of 0 to 2 cM (depending on the marker number of a group) in each part were removed. Then, the remaining markers from all the parts of a group were put together to generate a linkage map for the group. Closely linked markers with genetic distance of 0 to 2 cM were removed again. All markers with a genetic distance of 0 to 2 cM were put into a bin. Finally, when those previously removed markers were added to their corresponding bins, a genetic bin map was assembled.

### Matching Solexa sequences with SRAP markers

A SRAP profile produced by a pair of SRAP primers always included multiple fragments with different sizes. Each fragment theoretically corresponded to a specific locus in the genome. A Solexa paired-end sequence was located at the two ends of a SRAP fragment. To match Solexa sequences with their corresponding SRAP markers on the ultradense genetic map, locus-specific primers were designed using Solexa sequences (Additional file 3) [GenBank:SRA035245]. The ends of these Solexa paired-end sequences containing reverse primers were used in the primer designing. After the primers were removed from these Solexa sequence ends, the left sequence parts corresponded to specific loci and one sequence, to one locus in most cases in the genome were used for the locus-specific primers. These locus-specific primers were combined with the original forward primers to amplify the SRAP products obtained from sixteen RI lines and the original SRAP forward and reverse primers in the mapping procedure. The PCR set-up and running program was the same as the tagging PCR described previously. These labelled locus-specific PCR products were separated with the ABI 3100 genetic analyzer. The fragments and their sizes were scored and compared with the SRAP markers that were generated with the corresponding SRAP primers.

### Direct integration of Solexa sequence data on the genetic map

The second and third set of SRAP primers were used to amplify 94 DNA samples for the whole mapping population and SRAP PCR products were tagged with two sets of tagging primers to distinguish the DNA samples. Therefore, it was possible to integrate Solexa sequences onto the SRAP genetic map on the basis of Solexa sequence frequencies in each DNA sample. First, all tagging primer and SRAP primer sequences were eliminated from the Solexa paired-end sequences to retain the sequences that corresponded to the sequenced genomic parts. Then, each unique trimmed paired-end sequence was searched and counted in the previously established ninety-four files that corresponded to the ninety-two RI lines and the two parents of the mapping population. Finally, all unique trimmed Solexa paired-end sequences corresponding to individual loci in the genome and the absolute numbers of each trimmed unique Solexa paired-end sequences represented continuous variation of PCR amplification at a locus.

Statistical significance tests of linkage analysis between each unique Solexa paired-end sequence and 465 markers of the genetic bin map were carried out by Windows QTL Cartographer software 2.5 (http://statgen.ncsu.edu/qtlcart/WQTLCart.htm) using 'single marker analysis' function. The threshold was set at LOD score 2.5 (empiric QTL confidence intervals 99%). The unique Solexa sequences having significant linkage were assigned to the bin that had the maximum LOD score. Unique Solexa sequences which had no significant linkage to all bins or significant linkage to several bins were discarded. Thus, unique Solexa paired-end sequences were assigned into their corresponding bins on the genetic map after statistical significance testing. Compared with SRAP markers, some integrated Solexa sequences were identified as closely linked to SRAP markers that were produced with the same SRAP primers.

### Alignment of the current genetic map with other published genetic maps

There were 243 common SRAP primer pairs that were used to construct both the current genetic map in *B. rapa *and the previously published genetic map in *B. napus *[2]. Since 10 *B. rapa *chromosomes have their corresponding counterparts in *B. napus*, the same SRAP markers may exist in both genetic maps. All SRAP markers that were produced by the same SRAP primer pairs and which had similar fragment sizes were identified to align the current *B. rapa *genetic map with the previous *B. napus *genetic map. Additional SSR markers used previously were used to align the current map with other published ones.

## Results

### Construction of an ultradense genetic map and alignment of the current genetic map with the previous ones

**Figure 1:**
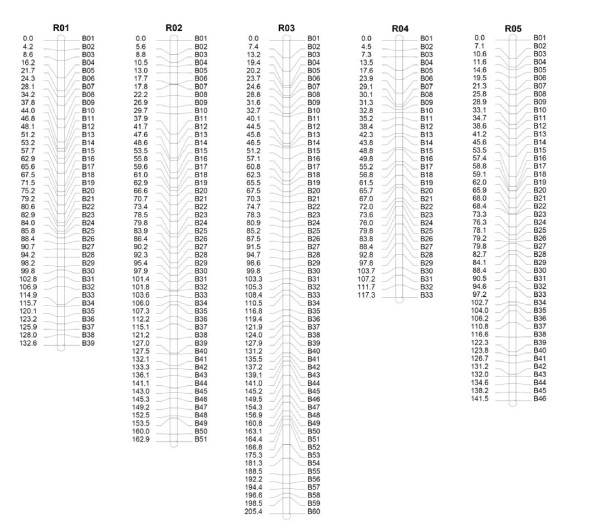
**Genetic bin map of *B. rapa *(R1-R5)**. The genetic distance (cM) was indicated on the left and the bin name on the right of each linkage group.

**Figure 2:**
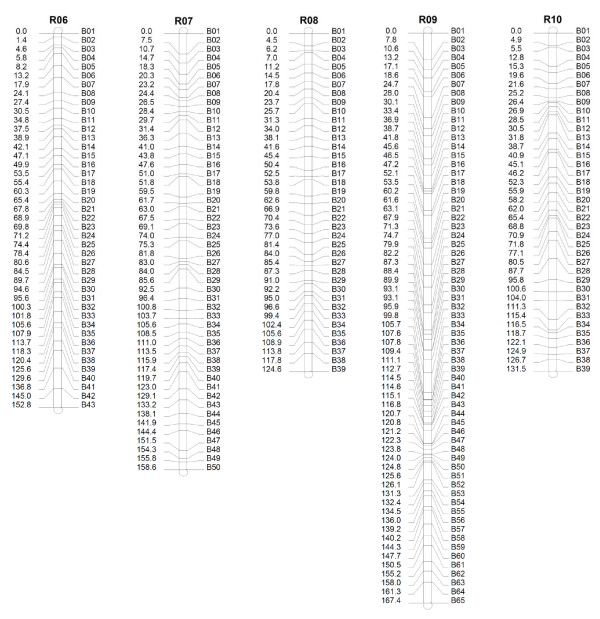
**Genetic bin map of *B. rapa *(R6-R10)**. The genetic distance (cM) was indicated on the left and the bin name on the right of each linkage group.

Eight hundred and five SRAP primer pairs were used to score 11,711 SRAP polymorphic loci. The average polymorphic locus per primer pair was 14.5. After analysis by JoinMap 3.0 software, 9,177 SRAP markers were assembled into 465 bins on 10 linkage groups with the LOD score from 10 to 12 (Additional file 4). The genetic bin map covered a genetic distance of 1,495.6 cM (Figure 1, Figure 2 and Table 1). Linkage group 9 containing 1,565 markers was the biggest group, while Linkage group 10 was the smallest group with 565 markers. The average number of SRAP markers in a bin was 19.7. There were 388 bins with ten or more SRAP markers, three bins with over 100 SRAP markers and only three bins with one SRAP marker, suggesting that the SRAP markers were evenly distributed in the *B. rapa *genome (Additional file 5).

**Table 1:** Summary of the ultradense genetic bin map of *B. rapa*.

Linkage group	No. of bins	No. of SRAP	No. of SSR	No. of Solexa sequences	Marker loci on each group	Length (cM)	No. of SRAP matched with sequences
R01	39	905	13	169	1087	132.6	16
R02	49	933	1	136	1070	164	19
R03	60	1293	4	248	1545	205.4	15
R04	33	573	2	128	703	117.3	5
R05	46	725	2	178	905	141.5	15
R06	43	949	2	181	1132	152.8	15
R07	50	874	9	129	1012	158.6	7
R08	40	795	5	148	948	124.5	16
R09	66	1565	2	301	1868	167.4	20
R10	39	565	6	119	690	131.5	13
total	465	9177	46	1737	10960	1495.6	141

There were 241 SRAP primer pairs that were used in both the current genetic map of *B. rapa *and the previous genetic map of *B. napus *[2]. Since the A genome in *B. napus *comes from *B. rapa *[20], the alignment of these two genetic maps would allow the identification of the corresponding linkages groups in these two related genomes. After comparison, it was found that 388 SRAP markers with size differences within two nucleotides on both the genetic maps were produced with the same SRAP primer pairs. When these SRAP markers were arranged on the basis of the bin order of each linkage group of the current map, one linkage group had the most closely matched SRAP markers of the *B. napus *genetic map was identified (Additional file 6). There were 10 common markers in R01 and N01, and the common markers in R02~R10 and N02~N10 were 12, 12, 9, 13, 15, 16, 5, 14 and 9 respectively. Thus, the ten linkage groups in the *B. rapa *genetic map were assigned R1 to R10 that corresponded to N1 to N10 in the genetic map of *B. napus*.

An alignment was also made with the published SSRs (Table 2). With the 30 previously used SSR primers [2,21-24], forty-six SSRs were developed and integrated on the current genetic map. Among these SSRs, 37 were found to have common linkage groups on the SSR genetic map reported by Piquemal et al. [24] and 22 on the Sun's genetic map [2]. A few to a dozen of these SSRs were also aligned with three other genetic maps.

**Table 2:** Alignment of the *B. rapa *linkage map with published genetic maps in *B. rapa *and *B. napus *by SSR markers

Primer name	Marker size	Map position	Choi (2007)	Kim (2006)	Cheng (2009)	Piquemal (2005)	Sun (2007)
BRAS041	352	R01b01				N1	N01
CB10099c	243	R01b09			A1	N1	N01
CB10099b	221	R01b14			A1	N1	N01
CB10099a	219	R01b18			A1	N1	N01
BRAS011d	227	R01b19					
BRAS002b	225	R01b22					
BRAS067b	275	R01b26				N1	
BRAS011e	299	R01b27					
Na14-F11e	263	R01b27				N1	
Na14-F11f	270	R01b31				N1	
BRAS067a	265	R01b39				N1	
BRAS067c	277	R01b39				N1	
Na14-F11a	246	R01b39				N1	
Na14-F11b	252	R01b39				N1	
Na14-F11c	256	R01b39				N1	
Na14-F11d	262	R01b39				N1	
CB10355	221	R02b51			A2	N2	
Na12-A01b	152	R03b07					
BRAS002a	202	R03b10					
Na12-E02a	118	R03b39	R03		A3	N3	N03
Na12-E02b	146	R03b39	R03		A3	N3	N03
Na12-E02c	151	R03b39	R03		A3	N3	N03
CB10036	151	R03b46				N3	N03
CB10347	205	R04b11					N04
BRMS129	289	R04b25					
CB10493	177	R04b29				N4	N04
CB10229a	268	R05b13					N05
CB10229b	271	R05b13					N05
ENA8e	463	R05b46					
CB10330a	166	R06b01				N6	
CB10330b	172	R06b01				N6	
Na12-A01a	143	R06b18					
BRAS011b	190	R06b28					
Na10-C01	439	R06b31					
Ra2-G08	320	R07b08			A7	N7	
ENA8b	312	R07b12	R07				
ENA8c	357	R07b12	R07				
ENA8d	351	R07b12	R07				
BRMS018a	176	R07b13			A7		
ENA8a	304	R07b15	R07				
CB10278a	230	R07b25				N7	
BRAS002c	229	R07b29					
BRMS018b	245	R07b31			A7		
CB10278b	243	R07b31				N7	
Ra2-E12b	190	R08b23	R08	R8		N8	N08
Ra2-E12c	192	R08b23	R08	R8		N8	N08
Ra2-E12a	189	R08b24	R08	R8		N8	N08
BRAS023a	205	R08b30					
BRAS023b	217	R08b30					
CB10364b	224	R08b30				N8	N08
BRAS011c	225	R08b36					
CB10364a	218	R08b38				N8	N08
BRAS011a	179	R09b36					
BRAS011f	328	R09b41					
OI10-D08	228	R09b43			A9		
Ra1-F06	257	R09b45					
Na10-A08	160	R09b59			A9	N9	N09
CB10124a	165	R10b01				N10	
CB10124b	174	R10b01				N10	N10
Na10-G08	153	R10b04				N10	
CB10524a	237	R10b20				N10	N10
CB10524b	240	R10b20				N10	N10
CB10524c	243	R10b25				N10	N10
CB10097	174	R10b32					

### Matching Solexa paired-end sequences with SRAPs in the first set of SRAP primers

To prepare a pooled sample for Illumina's Solexa sequencing, 77,568 SRAP PCR reactions were run in 202 384-well plates and the same number of tagging PCR reactions were performed in another 202 384-well plates. All products in the 77,568 tagging PCR reactions were pooled to form one sample for Illumina's Solexa sequencing. Three sets of SRAP primers were used to test the effectiveness and efficiency of combining SRAP with Illumina's Solexa sequencing and maximize the application potential of SRAP technology in genetic map construction and molecular marker development.

In the first set of SRAP reactions, 2,688 SRAP primer pairs from 14 forward primers and 192 reverse primers were used to amplify two DNA samples of the parental lines of the recombinant inbred (RI) line mapping population in 14 384-well plates. After analysis of Illumina's Solexa sequencing data, 69,836 paired-end sequences that were produced with the first set of SRAP primers in 14 384-well plates were identified which belonged to 11,272 unique Solexa paired-end sequences. Among these unique Solexa paired-end sequences, 9,693 had one to two paired-end sequences, 1473, three to nine paired-end sequences and 1167, 10 and more paired-end sequences respectively.

### Identification of Solexa paired-end sequences corresponding to SRAP markers on the ultradense genetic map

**Figure 3:**
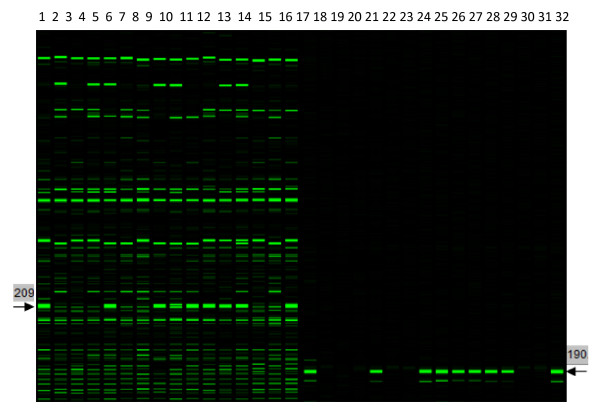
**Comparison of the SRAP profiles (lines 1-16) produced with SRAP primers EM1 (fluorescently labelled) and PM33 and the specific PCR profiles (lines 17-32) produced with SRAP primer EM1 (fluorescently labelled) and locus specific primer LW024**. The arrows showed the SRAP bands amplified with 16 DNA samples matched by the locus specific PCR bands amplified with the SRAP PCR products in lines 1 to 16. The numbers on both sides showed the band sizes indicated by arrows.

One of the major goals in this research was to identify Solexa sequences that correspond to SRAP markers so that these SRAP markers became characterized with partially known sequences. Five hundred and thirty-two Solexa sequences that were obtained with 165 SRAP primer pairs used in the genetic map construction were selected to design 532 locus-specific primers. In the locus-specific PCR, some locus-specific primers produced single fragments. When the scores generated with the locus-specific primers were identical to the scores of the SRAP markers generated with the corresponding original SRAP primers and the fragment sizes in the locus specific PCR were the expected sizes compared with the sizes of the matched SRAP markers, the Solexa paired-end sequences used for the locus-specific primers were assigned to the SRAP markers (Figure 3). As expected, the fragment sizes in the locus-specific PCR were about 20 nucleotides shorter than the original SRAP fragments since the sequences of SRAP reverse primers were removed during the locus-specific primer designing.

Out of the 532 locus-specific primers, 141 were assigned to their corresponding SRAP markers on the ultradense genetic map. Therefore, the corresponding 141 SRAP markers had partial known sequences. These 141 markers were evenly distributed on the 10 linkage groups (Table 1). There were 5 markers on R04 and 7 markers on R07, the other linkage group had 13 or more markers. However, 112 locus-specific primers had PCR products, but did not produce polymorphic loci, and other 277 locus-specific primers did not have good amplification (Additional file 3).

On the basis of the sequence counts, the Solexa paired-end sequences used for designing the 532 locus-specific primers were classified into three groups with 1 to 2, 3 to 9, and 10 or more counts, respectively. The results showed that the numbers of assigned Solexa sequences were quite different among these three groups. The Solexa paired-end sequences with successfully matched SRAP markers in these three groups were 16.0%, 32.5% and 46.6% respectively, indicating that unique Solexa paired-end sequences with a large number of sequence counts would be easier to assign to its corresponding SRAP markers (Table 3).

**Table 3:** Comparison of Solexa sequence counts and frequency of matched Solexa sequences with their corresponding SRAP markers tested with 532 specific primers

Counts of Solexa sequences	No. of sequences matched with SRAP	No. of tested sequences	Successful rate
1~2	47	294	15.99%
3~9	39	120	32.50%
>9	55	118	46.61%
total	141	532	26.50%

### Integration of Solexa paired-end sequences on the ultradense SRAP genetic map

In preparing the Illumina's Solexa sequencing sample, the second and third sets of SRAP primers were used to amplify 92 RI lines and two parents of the mapping population respectively and the tagging primers allowed the identification of the original SRAP products that belonged to the individual DNA samples. Ninety-four files with the second set of tagging primers and another 94 files with the third set of tagging primers were analyzed respectively and unique Solexa sequence paired-end sequences from the files that corresponded to the parental lines were identified. Each of these unique Solexa sequences was counted individually in the corresponding 92 files that were established for the 92 RI lines of the mapping population. After counting, 55,453 unique Solexa paired-end sequences that were generated with the second set of SRAP primers were identified. Similarly, 136,475 unique Solexa paired-end sequences generated with the third set of SRAP primers were identified. In total, 191,928 unique Solexa paired-end sequences in all 188 files were obtained. The unique Solexa paired-end sequences were not found in more than 70 RI lines and considered low frequency. After eliminating these Solexa paired end sequences with low frequencies, 2,172 unique Solexa sequences in the second set of SRAP primers and 4,243 unique Solexa sequences in the third set of SRAP primers were selected for the following analysis. QTL software was adapted to test linkage statistical significance of each unique Solexa paired-end sequences using the 465 bins on the genetic map. Among all 6,415 selected unique Solexa paired-end sequences generated with both the second and third sets of SRAP primers, 1,737 were integrated on the ultradense genetic map (Table 1, Additional file 7) [GenBank:SRA035245]. Among these 1,737 integrated groups, 651 belonged to the PCR products amplified with the second set of SRAP primers and the other 1086, the third set of SRAP primers. In contrast, 435 more integrated Solexa paired-end sequences in the third set of SRAP primers were obtained than those from the second set of SRAP primers.

All integrated Solexa paired-end sequences fell into 314 bins of the 465 bin map and all bins contain two or more sequences. The bin R9bin48 with 52 integrated Solexa paired-end sequences was the most concentrated one. There were another 38 bins with 10 or more integrated Solexa paired-end sequences and the rest of these bins had two or more integrated Solexa paired-end sequences. The distribution of the integrated Solexa paired-end sequences was similar to that of SRAP markers on the ultradense genetic map.

Among the 1,086 integrated Solexa paired-end sequences that were obtained with the third set of SRAP primers, 407 were produced with 76 SRAP primer pairs that were also used in the construction of the ultradense genetic map. One hundred and one of the 407 Solexa paired-end sequences fell into the bins or the neighbouring bins. Fifty-five Solexa paired-end sequences fell into the same bins with the SRAP markers that shared the same SRAP primers. These perfectly matched SRAP and integrated paired-end sequences suggested that these SRAP markers and their corresponding Solexa paired-end sequences come from the same loci (Additional file 8).

## Discussion

### SRAP is an adequate method for constructing ultradense genetic maps

Similar to the previous genetic map with 13,351 SRAP markers in an amphidiploid species *B. napus*, an ultradense genetic map in a diploid species *B. rapa *was constructed in this study. Using the SRAP method to construct two ultradense genetic maps was not difficult since one SRAP primer combination may detect over 10 polymorphic loci. With a medium throughput ABI 3100 Genetic Analyzer, it is feasible to assemble an ultradense genetic map with over 10,000 SRAP markers in a few months.

When SRAP PCR products are separated with the ABI Genetic Analyzer, it is feasible to obtain the fragment sizes of SRAP markers. Although the fragment sizes of SRAP markers may show one to two base differences due to the changing data collection conditions from time to time and variations related to scoring procedure with Genographer software, it is reliable to compare SRAP markers that are produced with the same primers within a species or even between related species in a genus. In this report, the alignment of two genetic maps was successfully performed to assign the linkage groups of the new genetic map with previous genetic maps. Moreover, there are 6 SRAP markers in R02 of *B. rapa*, while they are also found in N12 of *B. napus*. Common markers were also found between R03 and N13 (7 markers), R03 and N07 (7 markers), R03 and N09 (5 markers), R06 and N12 (5 markers), R09 and N11 (5 markers), R09 and N12 (6 markers), R09 and N19 (5 markers), R09and N05 (9 markers), R09 and N08 (13 markers). These results might be explained by the colinearity of the A and C genomes or the duplication of chromosomes within the A genome [2,23-26]. Currently, these two genetic maps are intensively used to make alignment of genetic maps that are generated with different mapping populations. For example, the *B. napus *genetic map is being used to align other five genetic maps that are used to perform QTL mapping of Sclerotinia tolerance in *B. napus *while the *B. rapa *genetic map is being used to align another genetic map that is being used for QTL mapping of glucosinolates in *B. rapa *in the lab (unpublished data).

SSR markers are commonly used to make alignment of genetic maps in Brassica species. Although it is common to amplify more than one locus in *B. napus*, it is possible to align linkage groups of different genetic maps. The previously used SSR were integrated in the current *B. rapa *genetic map to show the correct alignment of four genetic maps.

### Solexa paired-end sequences are assigned to their corresponding SRAP markers

Illumina's Solexa sequencing is able to produce millions of paired-end sequences and this feature allows the sequencing of thousands of SRAP PCR reactions in one run. Through sequencing of tagged SRAP PCR products of a RI line mapping population and their parents, 1,737 Solexa paired-end sequences were integrated in the *B rapa *genetic map. Locus-specific PCR methods allowed matching Solexa sequences with their corresponding SRAP markers and with the mapping population, Solexa sequences were integrated on the genetic map through linkage statistical significance tests by using QTL software.

Using Illumina's Solexa sequencing, it was possible to determine the partial sequences of SRAP markers. In a species where the whole genome sequence is available, Solexa sequences that are produced in a small mapping population allow anchoring SRAP markers in the genome. In a genome where the genome sequence is not available, the SRAP markers with known Solexa sequences on a genetic map might be used to assemble the whole genome sequence. Since SRAP possesses multiplexing features in PCR reactions, it is not difficult to assemble thousands of SRAP markers in a short time such as the previous *B. napus *genetic map [2] and the current *B. rapa *genetic map. With an ultradense genetic map and known SRAP marker sequences, the scaffolds obtained with next generation sequencing might be easily assembled.

The frequency of a unique Solexa sequence represents the amplification level of a specific locus in a genome. If the sequences of two alleles from two parental lines that are annealed by a SRAP primer pair are different, the amplification of these two alleles becomes differentiated. This polymorphic locus becomes detectable if the amplification efficiencies of two alleles in a SRAP PCR are different enough. In a SRAP profile, there are some strong and weak bands. In particular, the strong bands that correspond to the loci with strong amplification are generally scored accurately if the amplification of two alleles of a locus is quite different. However, there are more score errors for the weak bands in a SRAP profile due to the minor variations in PCR set up and separation of PCR products. In general, these markers with score errors are not easy to be assembled on a genetic map. In this report, 532 Solexa sequences were selected to design locus-specific primers used to assign Solexa sequences with their corresponding SRAP markers. If Solexa paired-end sequences occur in high abundance of the pooled sample, these sequences are relatively easily assigned to their corresponding SRAP markers (46.6% in this report). On the other hand, 53.7% of the locus-specific primers that have low counts of Solexa paired-end sequences (1 to 2 counts) did not amplify well in their PCR reactions. These solexa paired-end sequences should represent those weak bands in SRAP profiles.

### Linkage analysis allows integration of Solexa sequences on an ultradense genetic map

High throughput is common in next generation sequencing, which makes it possible to count thousands of unique SRAP sequences in a mapping population through sequencing of tagged SRAP PCR products. Since SRAP contains two random primers with a size of 15-25 nucleotides, it is feasible to combine one forward primer with unlimited reverse primers. Thus, it is easy to tag SRAP PCR products using a second round of tagging PCR since all SRAP PCR products share the same forward primer and a set of tagging primers are designed with the part of the forward primer. Each tagging primer is used to tag one individual DNA sample and a hundred to a few hundreds of DNA samples in a mapping population might be tagged with the same number of tagging primers.

**Figure 4:**
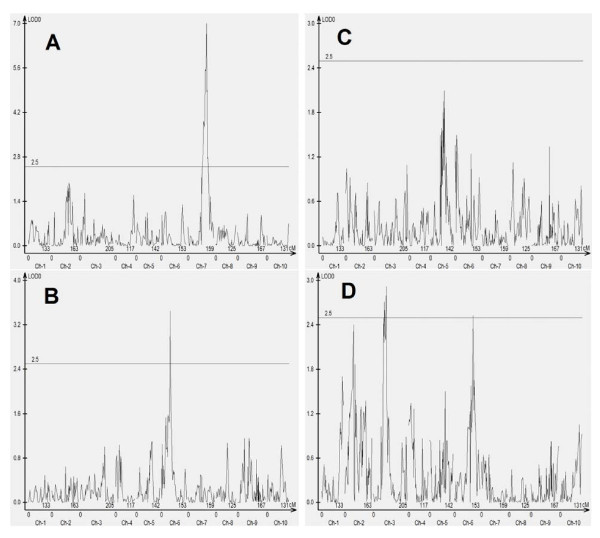
**Four examples in significance linkage testing with Windows QTL Cartographer**. A, Single linkage with high LOD value; B, Single linkage with low LOD value; C, No significant linkage; D, Multiple linkage with low LOD values. A and B were selected, and C and D were discarded.

After Illumina's Solexa sequencing, all sequence were sorted according to tagging primer sequences, which allowed the identification of original individual DNA samples where sequences were obtained. The frequencies of unique Solexa paired-end sequences in the tagged SRAP PCR products showed continuous variations among the individual RI lines of the mapping population, suggesting that two alleles of these polymorphic loci that corresponded to these unique Solexa paired-end sequences were amplified differentially in SRAP PCR. Polymorphic loci produce continuous distribution with real differences in sequence counts while monomorphic loci produce random continuous distribution in sequence counts. Therefore, when linkage analysis was applied, only these polymorphic loci detected as significant linkage were assigned into bins. The monomorphic loci with no significant linkage or showing significant linkage to several bins at low LOD values were discarded. Actually, in order to increase the accuracy of the results, only those sequences with high LOD scores or with low LOD scores but clean backgrounds were selected (Figure 4).

In this study, over 1,737 Solexa sequences were integrated in the genetic map through linkage analysis by statistical significance test. There were 76 common primer pairs that were used in both genetic bin map construction and Illunima's Solexa sequencing. Fifty-five integrated Solexa paired-end sequences and 55 SRAP markers produced with the same primer pairs were mapped into the same bins and these SRAP and integrated sequences matched with each other perfectly, suggesting that Statistical significance test by QTL software is an effective method to integrate Solexa sequences on a genetic map.

The diversity of SRAP primers affects the efficiency of integration of Solexa paired-end sequences on a genetic map. Two sets of SRAP primers were used to amplify the same RI line mapping population in this report. The results suggested that a high level of sequence similarity of SRAP primers reduces the number of detected unique Solexa paired-end sequences. In the second set of SRAP primers, all four forward primers had identical 12 nucleotides at the 5' end and so did all 96 reverse primers. This set of SRAP primers sharing a common sequence part were easily used for tagging SRAP PCR products and even two ends of SRAP PCR products in the second round of tagging PCR. However, it was found that a reduced number of detected SRAP products occurred in this set of SRAP primers. This set of SRAP primers produced fewer unique Solexa paired-end sequences than those produced with the third set of SRAP primers. As a result, the third set of SRAP primers in which one forward primer was combined with 384 different reverse primers in SRAP PCR maximized the detection of polymorphic loci. Therefore, this set of SRAP primers produced more integrated Solexa paired-end sequences than the second set of SRAP primers.

Random distribution of integrated Solexa paired-end sequences on a genetic map is essential to cover the whole genome when these integrated sequences are used to assemble sequence scaffolds into a whole genome sequence. The results in this report suggest that the integrated Solexa paired-end sequences that were produced with one forward primer and 384 reverse primers were quite evenly distributed on the genetic map of *B. rapa *and no clear clusters of SRAP markers on a genetic map were identified. Therefore, in a collection of different SRAP amplification reactions involving many primer pairs, one primer (as forward or reverse primer) might be the same and others (as reverse or forward primers) should be different to guarantee a random distribution of all SRAP markers in a genome.

## Conclusion

We constructed an ultradense genetic map in *B. rapa *using SRAP technology, which demonstrated that SRAP is relatively feasible to construct an ultradense genetic map with over 10,000 markers. Two methods were applied to match and integrate Solexa sequences on the genetic map and in total, 1,878 Solexa sequences fell into 416 out of 465 bins on the genetic map, suggesting that these Solexa sequences are evenly distributed in the genome. Currently, these integrated sequences are being used to improve the assembly of genome sequence scaffolds in *B. rapa *(in collaboration with Dr. Xiaowu Wang from CAAS, personal communication). Moreover, the sequence integration method might be used in other species where the genome sequencing has not been finished yet. The ultradense genetic map was aligned with other Brassica genetic maps and this map will be useful to the Brassica community to develop molecular markers and clone genes of interest.

## Authors' contributions

WL carried out genetic map construction, Solexa sequence integration and participated in developing SRAP markers and drafting the manuscript. JFZ performed DNA preparation for sequencing, and participated in SRAP marker development. YLM worked in the Solexa raw data analysis and interpretation. JFG developed mapping population and SSR markers. PBEM and SWH participated in the preparation of the manuscript. GL designed the whole research and participated in the preparation of the manuscript. All authors read and approved the final manuscript.

## Funding

This work was supported by the Genome Canada/Genome Alberta and Genome Prairie and the Manitoba Provincial Government, and the Canola Council of Canada and NSERC. Wei Li was also supported by the China Scholarship Council.

## Supplementary Material

Additional file 1 **Ninety-two F7 RI lines and the parental lines used in the ultradense genetic bin map construction**. Click here for file

Additional file 2 **SRAP and tagging primers**. Click here for file

Additional file 3 Locus specific primers, their original Solexa sequences, matched SRAP markers and bin position on the genetic map of *B. rapa.* Click here for file

Additional file 4 **9,177 SRAP markers with the corresponding SRAP primers, Sizes of SRAP PCR products and map positions**. Click here for file

Additional file 5 Distribution of SRAP, SSR, characterized SRAP and mapped Solexa sequence markers on the genetic map in *B. rapa.* Click here for file

Additional file 6 **Alignment of SRAP markers on the genetic map of *B. rapa *and the previous genetic map of *B. napus *(Sun et al. 2007)**. Click here for file

Additional file 7 Integrated Solexa sequence loci on the ultradense genetic bin map of *B. rapa.* Click here for file

Additional file 8 **Comparison of read numbers of some integrated Solexa sequences and scores of SRAP markers that were produced with the same primers and have the same positions on the genetci map of *B.rapa***. Click here for file
